# ATNet: A Defect Detection Framework for X-ray Images of DIP Chip Lead Bonding

**DOI:** 10.3390/mi14071375

**Published:** 2023-07-05

**Authors:** Renbin Huang, Daohua Zhan, Xiuding Yang, Bei Zhou, Linjun Tang, Nian Cai, Han Wang, Baojun Qiu

**Affiliations:** 1 School of Mechanical and Electrical Engineering, Guangdong University of Technology, Guangzhou 510006, China; 2112101249@mail2.gdut.edu.cn (R.H.); zhandaohua@mail2.gdut.edu.cn (D.Z.); 2112101016@mail2.gdut.edu.cn (X.Y.); 2112101245@mail2.gdut.edu.cn (B.Z.); 2112101486@mail2.gdut.edu.cn (L.T.); 2 School of Information Engineering, Guangdong University of Technology, Guangzhou 510006, China; cainian@gdut.edu.cn; 3 China Electronic Product Reliability and Environmental Testing Research Institute, Guangzhou 511370, China

**Keywords:** chips, defects, deep learning, X-ray images

## Abstract

In order to improve the production quality and qualification rate of chips, X-ray nondestructive imaging technology has been widely used in the detection of chip defects, which represents an important part of the quality inspection of products after packaging. However, the current traditional defect detection algorithm cannot meet the demands of high accuracy, fast speed, and real-time chip defect detection in industrial production. Therefore, this paper proposes a new multi-scale feature fusion module (ATSPPF) based on convolutional neural networks, which can more fully extract semantic information at different scales. In addition, based on this module, we design a deep learning model (ATNet) for detecting lead defects in chips. The experimental results show that at 8.2 giga floating point operations (GFLOPs) and 146 frames per second (FPS), mAP_0.5_ and mAP_0.5–0.95_ can achieve an average accuracy of 99.4% and 69.3%, respectively, while the detection speed is faster than the baseline yolov5s by nearly 50%.

## 1. Introduction

There are three main stages in the production of chips: design, manufacturing, and packaging and testing [1,2]. Among them, testing is an important part that determines whether the chip can be put into the market or not. The frequent start and stop of electronic devices can aggravate the thermal cycling stress of the chip [3], which can lead to structural deformation, lead breakage, and even solder joint failure [4]. The defects, such as missing and redundant chip solder joints, interconnections, and lead bonding fractures, are the main components of the testing session. Detecting defects in chips is of great importance to improve the quality of chips and reduce production costs.

In order to better ensure the quality of the chip, a number of researchers and scholars have conducted a lot of research on lead bonding defect detection. Pecht et al. [5] used an electromagnetic resonance technique instead of the conventional lead bond tension test to detect the quality of lead bonding. Luo et al. [6] obtained the vibration of the wire by micro force sensors and interpreted the vibration signal in time, frequency, and phase domains to determine the integrity of the lead bonding. Feng et al. [7] evaluated bond quality using a time-frequency analysis of the electrical signal at the bond obtained from the ultrasonic generator. Kannan [8] proposed a technique based on the forced-resonance principle for detecting multilead bonding. However, these physical inspection methods are only applicable to the inspection before the chip is packaged, and new defects may be generated during the packaging process, so the chip needs to be reinspected after packaging.

The main defect of a packaged chip lies in the internal connection condition, so nondestructive X-ray inspection testing technology [9] should be used to reconstruct the internal image of the chip for defect determination using image reconstruction technology [10]. In actual production, since relying on the naked eye to discriminate X-ray chip images leads to low efficiency and a high error rate [11]; the experience, energy, and emotion of the workers can seriously affect the judgment results. In recent years, automatic vision-inspection technology (AVI) has been widely used in semiconductor defect inspection in order to reduce manual misjudgment. Sreenivasan et al. [12] determined solder joint quality based on the shape, size, and location of the solder joint. Huang et al. [13] introduced switching median filtering in the Canny algorithm, and the results showed a more pronounced edge detection of lead, along with better denoising. Perng et al. [14] were the first to propose a combination of image processing techniques and lead bonding simulation for the automatic detection of multilayer IC wire bonding locations. Lin et al. [15] proposed a spatial convolutional attention mechanism (SCA) and designed a lightweight mobile network that performs well in lead defect detection. Chan [16] combined machine learning, support vector machine (SVM,) and Hoff circle transform algorithms to detect the quality of bonded balls, but manual assistance is required to achieve higher accuracy. Xie [17] employed the utilization of 3D point cloud technology to identify lead bonding regions. They converted the extraction process into a point cloud classification problem, resulting in the precise categorization of lead bonding defects. Kao et al. [18] have developed a deep-learning-based solution aimed at detecting the improper installation of wire bonding headers in wire bonding equipment, resulting in significant cost reduction in manufacturing. Chen et al. [19] proposed a data-driven approach consisting of data preprocessing, feature engineering, and classification and combined this with neural networks to detect the quality of lead bonding. Although computer vision detection methods have been widely applied to wire bonding detection, there are still several significant issues that cannot be ignored: (a) Traditional machine vision detection is slow and requires the design of different feature extraction algorithms to extract various defect features; (b) there are multiple defect types and complex target backgrounds that severely affect detection accuracy; (c) some current deep learning network models have enormous computational and parameter requirements, placing high demands on computer performance; (d) the inference speed of certain models fails to meet the requirements. Therefore, this paper proposes a convolutional neural network-based approach with higher speed and more accurate and lighter performance to implement in a network for detecting lead bonding defects inside a chip.

Based on the above problems in this paper, we propose the ATSPPF module, which can better fuse multi-scale features and can, at the same time, be adaptively weighted according to the contributions to space and channels so as to extract more useful feature information. In order to better detect lead bonding defects, we constructed a network with faster detection speed and higher accuracy compared to yolov5 based on the proposed ATSPPF module. We used X-ray equipment to obtain the internal lead bonding defect images of defective chips and then trained the constructed dataset (WBD) on the network model proposed in this paper. The network we designed stands out for its exceptional speed and accuracy in comparison to existing advanced detection methods. The main components and highlights of the paper are summarized as follows:

1. A novel ATSPPF module is introduced, which enables comprehensive feature extraction. This module effectively combines features from various scales and employs adaptive weighting in both the spatial and channel domains to enhance the expression of features;
2. Based on the ATSPPF module, an accurate and fast chip wire bonding defect detection model framework, ATNet, was specifically designed to achieve the automated, rapid, and highly accurate detection of lead bonding defects;
3. Within the dataset, the average detection accuracy (mAP_0.5_) achieved was an impressive 99.4%, while the detection speed reached 146 frames per second (FPS), outperforming other state-of-the-art networks, such as yolov5s and yolox.

## 2. Related Work

### 2.1. Data Acquisition and Augmentation

**Figure 1:**
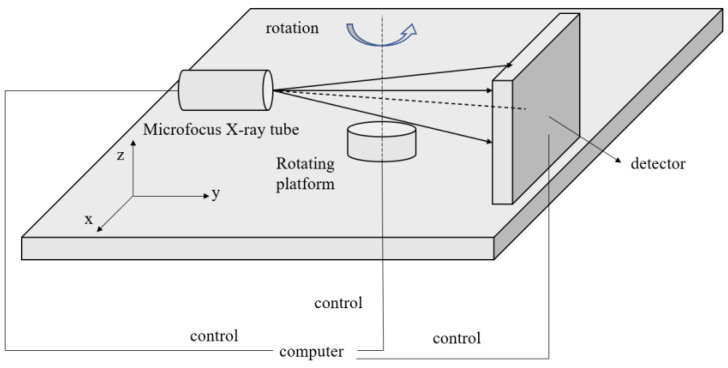
Schematic diagram of the CT equipment.

Due to the special nature of chip inspection, CT equipment is used to obtain the internal defects of the chip. As shown in Figure 1, the chip is placed on a rotating platform, and X-rays pass through the chip; the X-ray intensity will be attenuated to different degrees, and the detection panel will receive the attenuated X-ray intensity. Finally, by leveraging the intensity information of the rays, an X-ray image is reconstructed to depict the internal structure of the chip accurately. Pre-processing the obtained images is an important step to improve the correct rate of detecting lead bonding defects. In order to obtain better image quality and reduce the effect of noise on subsequent detection, image denoising is performed on the acquired images. In this paper, we first implement a typical method based on a median filter to remove the pepper noise from the image. With this nonlinear filtering process, the noise caused by various signal transmission errors can be well removed, while the edge information of the lead is well preserved. The wire part of the image is subsequently cropped to obtain the region of interest (ROI), which can largely reduce the influence of the background on the recognition results.

**Figure 2:**
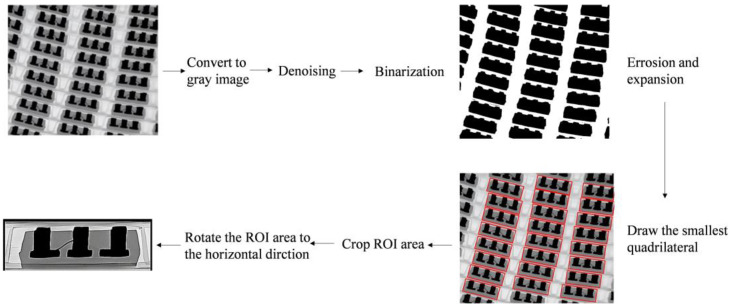
Shows the image preprocessing section and the extraction method flow for obtaining ROI prefetching.

As shown in Table 1, there are five types of lead chip bonding defects in this paper, namely: high loop, low loop, broken wire, sagged wire, and missing wire. Sample images of these defects and the process of extracting ROI regions are shown in Figure 2. The amount of data has a great impact on network training. Data expansion can make the network have better generalization and robustness and can prevent the network from over-fitting. Generally, image data can be expanded by random scaling, stretching, shearing, rotating, changing transparency, brightness, and other operations. In addition, some other scholars have proposed more unique data-enhancement methods, such as Mixup [20], Mosaic [21], and CutMix [22]. Therefore, to enhance the network’s generalization and robustness, the random combination of random scaling, cutting, rotating, and changing transparency and brightness is used to expand the data of the obtained image, and a set of lead defect data (Dataset-WBD) is constructed for training the network.

### 2.2. Object Detection

Currently, the majority of target detection networks can be categorized into three components: the backbone, neck, and detection head. The backbone part is used to extract the semantic features of the image; the neck part is to operate the features extracted at different stages of the backbone to make better use of the feature map, and the detection head is to detect the predicted object and the specific location of the object.

Backbone: The selection of an appropriate backbone network plays a crucial role in determining the overall performance of the network, so the selection of a backbone should conform to the characteristics of the dataset. At present, the main backbone networks include ResNet [23], MobileNet [24], Darknet 53 [25], Swin Transformer [26], VGG [27], etc. These backbone networks have fully verified their advantages in feature extraction in many experiments. On the basis of these backbone networks, researchers have fine-tuned them as their own backbone networks so as to make them more suitable for their own datasets.

Neck: Early networks, such as VGG, AlexNet [28], and Resnet, only used simple convolution operations to predict the final layer of the feature extraction network. However, the deep network is not sensitive to small target objects, so it often has no good effect on detecting small target tasks. In order to effectively avoid this problem, Lin et al. [29] developed an architecture with a horizontal connection and a top-down channel (FPN structure), which is used to fuse adjacent feature maps, make information flow between adjacent layers, form multi-scale feature maps, and enhance feature extraction. Szegedy et al. [30] pointed out that more network branches would reduce the parallelism of the model. Therefore, the recognition speed of the network will be reduced to a certain extent after the introduction of the FPN structure, but the detection accuracy will be greatly improved. Liu et al. [31] introduced a new bottom-up aggregation path based on the FPN structure, which enhances and shortens the information path between the top and bottom layers. In addition to the several different neck layer structures and methods mentioned above, there are also methods of path aggregation, such as BiFPN [32] and ASFF [33].

Head: This part is to recognize the feature map of the target extracted from the Neck part. At present, there are two main head detectors: one-stage and two-stage. The one-stage target detector directly predicts the feature map, while the two-stage detection is different from the one-stage detector. It utilizes the region proposal network (RPN) to select candidate regions on the feature map and then identifies and locates the candidate regions. The two-stage detector can generally obtain more accurate results, but the detection process requires the use of the RPN network to select a specific area, which can lead to a slow detection speed. The one-stage detector offers a faster detection speed compared to the two-stage detector, but it may result in relatively lower detection accuracy.

### 2.3. The Position of the Target Object

In yolov5, each grid in the feature map of the prediction head section generates three prediction frames of different scales to predict the exact location of the target, so an object in the original map is covered by multiple prediction frames at the same time. In order to select the most suitable predictor box, the box with the largest intersection-to-parallel ratio (IOU) is chosen as the predicted target object location. However, there is no intersection between the real frame and the prediction frame, which will result in a loss of 0. The gradient cannot be backpropagated, and training is not possible. Moreover, since the real frame will have a certain tilt angle, it will lead to the inability to select the most suitable prediction frame with the same intersection ratio. Rezatofighi [34] proposed the concept of GIOU on the basis of IOU, which is used to calculate the minimum area of the region that encloses the real frame and the predicted frame and then calculate the proportion of the region that does not belong to the two frames in the region, and finally subtract this proportion from IoU to get GIoU. It can not only well reflect the overlap between the real frame and the predicted frame part but can also pay more attention to the non-overlapping part of the region, which can better reflect the overlap between the two. Zheng et al. [35] proposed DIOU, with a faster convergence rate, which can directly minimize the distance between two target frames and provide direction for the movement of the prediction frame. Meanwhile, Zheng introduced a weight parameter to extend DIOU and propose CIOU. This modification aims to address the issue of varying width and height between the predicted frame and the actual frame, thereby optimizing the overall performance. In order to optimize the training results, CIOU is used as the position loss of the prediction frame in this paper. The equation for computing CIOU can be represented as follows.

(1) $${CIOU}_{dist}=IOU-\left(\frac{\rho^{2}\left(box_{1},box_{2}\right)}{c^{2}}+\alpha \upsilon \right),$$

where $\rho^{2}\left(box_{1},box_{2}\right)$ denotes the Euclidean distance between the centroid of $box_{1}$ and $box_{2}$. *c* denotes the diagonal distance of the smallest closed region that can contain both $box_{1}$ and $box_{2}$. In Formula (3), $\omega^{gt},h^{gt}$, ω, and h represent the width and height of the real frame and the width and height of the prediction frame, respectively.

The formulas for *α*, *υ*, and ${LOSS}_{CIOU}$ are as follows.

(2) α=v/(1−IOU+v),

(3) $$v=\frac{4}{\pi^{2}}{\left(arctan\frac{\omega^{gt}}{h^{gt}}-arctan\frac{\omega }{h}\right)}^{2},$$

(4) $${LOSS}_{CIOU}=1-IOU+\frac{\rho^{2}\left(box_{1},box_{2}\right)}{c^{2}}+\alpha v,$$

### 2.4. Activation Functions

In artificial neural networks, the activation function plays a very important part, similar to the model of neurons in the human brain, where the activation function ultimately decides what to send to the next neuron. Its main role is to add a nonlinear operation to all implicit and output layers, enhancing the intricacy and expressiveness of the neural network’s output. The commonly used activation functions are Sigmoid, Tanh, ReLU, LeakyReLU, SELU, and SiLU. In this paper, we mainly use two activation functions, LeakyReLU and SiLU, for which the expressions and corresponding first-order $f^{\prime }(x)$ derivatives are as follows.

The SiLU activation function is expressed as follows, where σ(x) is the sigmoid activation function, and *x* is the input:

(5) f(x)=x·σ(x),

(6) $$f^{\prime }(x)=f(x)+\sigma (x)(1-f(x)),$$

The LeakyReLU activation function, where α is a constant with a value of 0.01:

(7) $$f(x)=\left\{\begin{array}{ll} \alpha x & x<0\\ x & x>0 \end{array}\right.,$$

(8) $$f^{\prime }(x)=\left\{\begin{array}{ll} \alpha & x<0\\ 1 & x>0 \end{array}\right.,$$

## 3. Methodology

**Figure 3:**
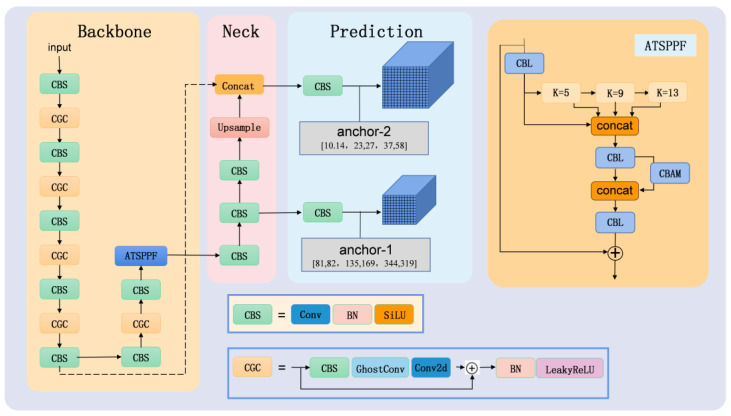
Schematic diagram of the ATNet network structure.

The Yolov5 framework utilizes C3 as the backbone layer, which enables it to recognize more intricate features. In this study, we propose the CGC module, building upon the structure of C3. Additionally, we introduce a novel feature fusion method called ATSPPF. This method adaptively assigns weights based on the contributions from spatial and channel dimensions, enhancing the network’s sensitivity to valuable spatial and channel information. By incorporating these advancements, the network becomes more proficient at identifying lead bonding defects across multiple scales. Yolov3-tiny, known for its fast detection speed due to its low computational requirements and simple network structure, excels in swiftly detecting simple features in industrial settings. Hence, we combine the network architecture of yolov3-tiny with the CGC module and ATSPPF module to propose ATNet, a network specifically designed for lead bonding defect detection. Figure 3 illustrates the structure of ATNet. Detailed descriptions of the CGC module and ATSPPF module proposed in this study will follow.

### 3.1. CGC Module

The C3 structure consists of three convolutional operations followed by a bottleneck layer. On the other hand, the CGC module modifies the bottleneck layer in C3 by substituting it with Ghostconv. Additionally, one of the convolution operations in the branch is eliminated and replaced with a residual connection. Experiments in Dataset-WBD showed that the use of the CGC module could improve mAP_0.5_ and mAP_0.5–0.95_ by 0.5% and 1.5%, respectively, compared to the use of the C3 module.

**Figure 4:**
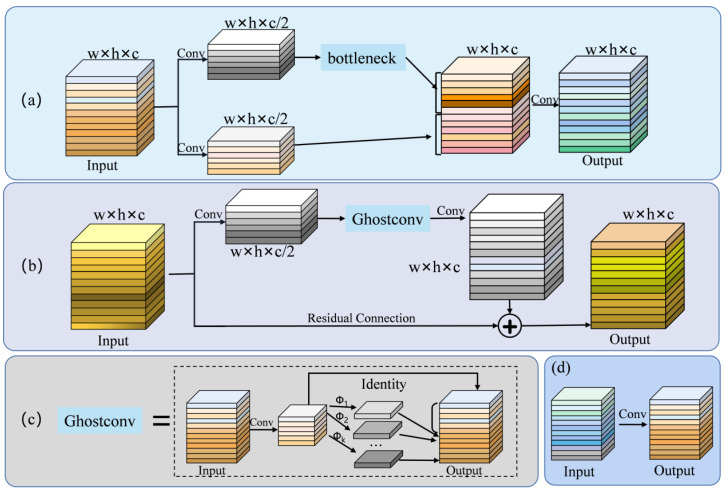
Comparison between the C3 module and CGC module: ghost convolution and standard convolution (**a**) C3 module, (**b**) CGC module, (**c**) ghost convolution, denotes a low-cost linear transformation procedure), and (**d**) standard convolution.

Han et al. [36] pointed out that due to the process of feature extraction by the network, a significant portion of redundant and duplicated features can occur if the number of channels is too large. Therefore, to achieve a better balance between extracting enough features and computational cost, the Ghostconv module was proposed. When compared with standard convolution, ghost convolution results in a substantial decrease in both computational workload and parameter quantity and the result of feature extraction is not much different from normal convolution. In this paper, we detail how the CGC module is constructed based on adopting Han’s similar idea. As illustrated in Figure 4, the CGC module uses a residual structure. Firstly, the number of channels will be reduced to c/2 using a standard convolution of 3 × 3. The result is input into Ghostconv, and then, by utilizing a 1 × 1 convolution, the number of channels is expanded to align with the dimensions of the original input feature map. This enables the addition operation to be carried out between the original input feature map and the resultant feature map. The inclusion of the residual connection guarantees the stability of the network.

In order to address the problems of gradient vanishing, gradient explosion, and overfitting that may be caused by the network depth being too deep, a residual connection is introduced into the CGC module, and the network’s capacity for learning is enhanced at the same time. The residual connection can be represented by the following equation. Where $H_{l}$ denotes the input, $H_{l+1}$ denotes the output, the nonlinear variation of the input is defined as *F*(*x*,*w*), and *w* denotes the weight parameter of the function *F*.

(9) $$H_{l+1}=H_{l}+F(x_{l},w,b),$$

By incorporating the Ghostconv module and residual structure into the CGC module, several benefits are achieved. These include significant reductions in computational requirements, the acquisition of ample features, and ensuring network stability.

### 3.2. ATSPPF Module

**Figure 5:**
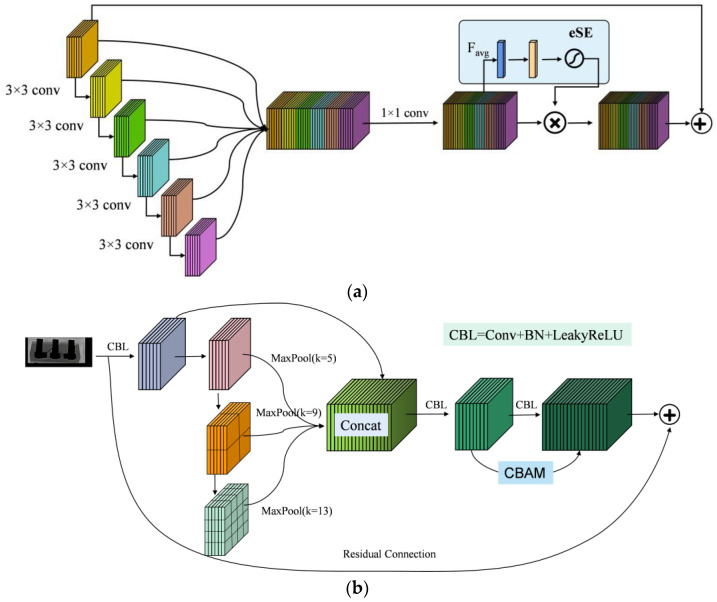
(**a**) The structure of the OSA + residual + eSE module; (**b**) the structure of the ATSPPF module.

VoVNet [37] incorporates the one-shot aggregate (OSA) module as its backbone, which enhances both the recognition speed and accuracy of the network when compared to the baseline DenseNet. Lee et al. [38] introduced residual connectivity and an effective squeezed excitation attention module (eSE) based on OSA, which avoids performance degradation due to the depth of the network being too deep while using eSE to further enhance the features. Inspired by this, a new spatial scale fusion module (ATSPPF) is proposed in this paper, and the specific structure diagram is shown in Figure 5 below. After performing standard convolution operations on the input features, we utilize three maximum pooling operations with kernel sizes of 5 × 5, 9 × 9, and 13 × 13 to extract feature information at various scales. These pooling operations ensure that features across different scales are preserved. The resulting feature maps from convolution and pooling are then stacked along the channel dimension to avoid any loss of feature information. Finally, a standard 1 × 1 convolution is employed to reduce the number of channels from 4C to C.

For the target detection task compared to the classification task, the size of the features to be recognized varies, so using feature maps of different scales can be more beneficial for detection. In order for the network to enhance its focus on the valuable feature channel information and spatial information and selectively ignore some less important feature channels, the CBAM attention mechanism module can be introduced at the end in parallel. Then, the number of channels of 2c is again transformed into c for subsequent residual connections, and finally, the residual connections are used to ensure the stability of the network. Table 2 presents the detailed structure of the ATSPPF module.

### 3.3. Feature Map

**Figure 6:**
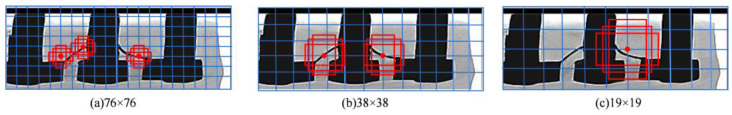
Schematic diagram of the size of the prediction boxes generated on different feature maps in Yolov5 in the original image.

Different feature maps have different sensory receptiveness and different sensitivities to the same size lead bonding defects. Yolov5s eventually predicts feature maps of 19 × 19, 38 × 38, and 76 × 76 in size. Among them, the 76 × 76 feature map is more suitable for detecting small targets, while for medium and large targets, feature maps of 19 × 19 and 38 × 38 in size are more effective. As illustrated in Figure 6, the feature maps are 76 × 76, 38 × 38, and 19 × 19 from left to right scale, respectively. For the 76 × 76 feature map, the resulting anchor box is too small to completely contain the defective parts, while for the 19 × 19 and 38 × 38 feature maps, recognition is better. Therefore, in order to reduce the complexity and computational effort of the network, we use feature maps of 13 × 13 and 26 × 26 in size based on yolov3-tiny. There are three different feature scales and corresponding anchor sizes of yolov5s. In Figure 6, the sizes of the anchors are not in proportion to the real anchor frame size, and this just wants to express that the different sizes of anchors will affect whether the objects can be detected.

Architecture: Based on the CGC and ATSPPF modules, this paper proposes an ATNet network with a strong feature-extraction ability and detection speed. The ATSPPF module is integrated at the end of the backbone network to facilitate the processing of significant information and bring the enhanced features closer to the output layer, resulting in improved accuracy for recognition outcomes.

## 4. Experiments

### 4.1. Experimental Setup

We implement our net using PyTorch deep learning framework and train it on an NVIDIA GeForce RTX 3050 graphics card 4G and an Interl 2.30GHz i7-11800H CPU. Dataset-WBD is divided into a train set and a test set according to the proportion of 90% and 10%. The training set is employed to optimize network parameters by minimizing the loss function, while the test set is utilized to assess the trained network’s accuracy in detecting wire bonding defects. The network is trained with the Stochastic Gradient Descent (SGD) optimizer with a linear decay learning rate scheduling strategy, in which the learning rate is initially set to 0.01 and gradually reduced to 0.001. We specify the batch size as 8, the momentum parameter as 0.937, and the weight decay rate as 0.0005. The input image is uniformly transformed to 640 × 640 size and normalized. The anchor dimensions were set to [10, 14], [23, 27], [37, 58] on the feature map of p/16 and [81, 82], [135, 169], [344, 319] on the feature map of p/32.

### 4.2. Evaluation Criterion

In the task of chip defect target detection, the Intersection over Union (IOU) is employed to assess whether a detected result corresponds to a true defect. If the IOU value surpasses the predetermined threshold (usually set at 0.5), it is classified as a positive sample; otherwise, it is regarded as a negative sample. In the target detection task, precision and recall are crucial metrics for evaluating the performance of network recognition, which are defined as follows:

(10) Precision=TP/(TP+FP),

(11) Recall=TP/(TP+FN),

where TP (true positive) refers to the number of true defects detected; FP (false positive) is the number of incorrectly predicted defects, which are not defects; and FN (false negative) indicates the number of actual defects missed. Note that (TN) (true negative) indicates the number of samples correctly predicted to be negative. For the prediction frames detected by the network, the discrimination between TP or FP is determined by the ratio of the overlap area between the prediction box and the true box, which is defined as follows.

(12) $$\gamma =area\left(A\cap B\right)/area\left(A\cup B\right),$$

where γ is the overlap ratio, A∩B represents the intersection area between the true box and the predicted box, and A∪B represents the total area between them. It will be set to 0.5 in this experiment. ${mAP}_{0.5}$ refers to the average accuracy when the IOU is 0.5, and ${mAP}_{0.5–0.95}$ refers to the average accuracy of 0.5 in increments of 0.05 to 0.95. In this paper*,* ${mAP}_{0.5}$ and ${mAP}_{0.5–0.95}$ are used to evaluate the indicators for judging the comprehensive ability of all categories and choose the time complexity GFLOPs and the space complexity parameters to represent the differences between the different methods.

### 4.3. Ablation Studies

We utilized ablation experiments to assess the benefits of the CGC module and the ATSPPF module, which were introduced in this paper, on the ATNet network. The outcomes of the experiments are presented in the following Table 3. Experiment 2 is 0.1% and 1% higher than Experiment 1 in ${mAP}_{0.5}$ and ${mAP}_{0.5–0.95}$, respectively, while the detection speed is also improved by nine frames. In experiment 3, after adding the ATSPPF module, the ${mAP}_{0.5–0.95}$ is 1.8% higher compared to experiment 1. In experiment 4, after introducing both CGC module and ATSPPF module, the ${mAP}_{0.5}$ and ${mAP}_{0.5–0.95}$ can reach 99.4% and 69.4%, respectively, and the detection speed can reach 146 (FPS). In order to detect wire bonding defects in real time and accurately in production, the combination of comprehensive comparison experiment 4 is more consistent with the requirements. The attention mechanism is incorporated into the ATSPPF module.

At present, three attention mechanism modules are commonly used: SE, CA, and CBAM. As shown in Table 4, after introducing three different attention mechanism modules into the ATSPPF module, the CBAM detection yields the highest result among the three options. Therefore, we use CBAM as the attention module in ATSPPF.

### 4.4. Comparison of State-of-the-Art Models

**Figure 7:**
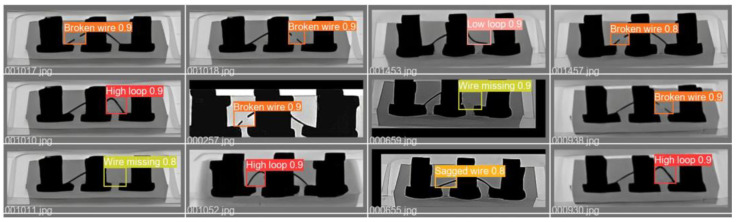
The detection result of our method on the test dataset of Dataset-WBD.

Here, we benchmark our approach against state-of-the-art defect detection methods. Table 4 lists the details of different performance indicators (mAP_0.5_, FPS, GFLOPs). It can be seen from Table 5 that the method proposed in this paper has great advantages in precision and detection speed compared with other methods. The detection results can be guaranteed that mAP_0.5_ reaches 99.4% when the detection speed is 146 FPS. For Faster R-CNN-ResNet50, Dynamic RCNN-ResNet50, and RetinaNet-ResNet50, their detection speed is less than 30 FPS, which is far from the requirement of real-time wire bonding detection. Due to their great GFLOPs, these methods also have high hardware requirements. Compared with YOLOv3-tiny, which has a similar network structure, the detection accuracy and speed of both are similar, but our method is much lower than YOLOv3-tiny in terms of both model size and computational complexity (GFLOPs), and the detection speed is nearly 50% higher than the baseline yolov5s. Our method has demonstrated its effectiveness in detecting defects, as illustrated in Figure 7. The detection results of our approach on the test dataset of Dataset-WBD are depicted.

**Figure 8:**
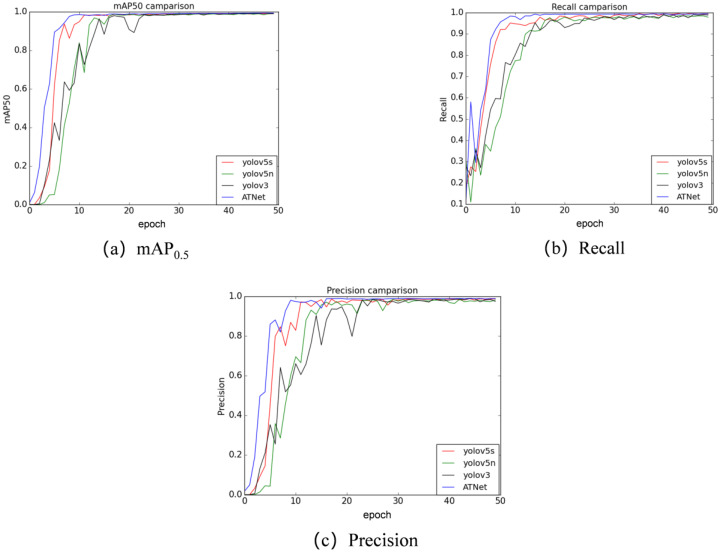
Convergence speed of different indexes: (**a**) mAP_0.5_; (**b**) Recall; (**c**) Precision.

In addition, in order to validate the effectiveness of the ATSPPF module, the module was introduced into the backbone tail part of other networks for training, and the results are shown in Table 6. From the table, it is apparent that the introduction of the ATSPPF module improves the Recall and mAP_0.5–0.95_ of the yolov3 network by 0.8% and 1.1%, respectively. In Faster-RCNN, although there is a small decrease in Recall, the accuracy is improved by 0.3%, and both mAP_0.5_ and mAP_0.5–0.95_ have a larger improvement. For different SSD backbone networks, almost four indicators have been improved to varying degrees. In addition, for the three networks, YoloR [39], LFyolo [40], and Sclaed_yolov4, the overall comprehensive detection performance has also been improved by introducing the ATSPPF module at the end of the feature extraction network. This shows that ATSPPF uses a pooling operation to fuse receptive field with different scales and introduces an attention mechanism, which can extract multi-scale information of the image, making the feature map have more abundant expression ability, thus effectively improving the detection performance of the model. In the network architectures of YOLOv5 and YOLOv3, both models incorporate three detection heads. This necessitates the network to allocate additional time for positional regression of targets detected by different prediction heads. However, it is possible that an object may be detected simultaneously by two detection heads, significantly impacting the convergence speed of the network. Therefore, as depicted in Figure 8, it can be observed that ATNet, which incorporates two detection heads, achieves faster convergence rates than YOLOv5 and YOLOv3 across three metrics: mAP_0.5_, recall, and precision. This also reflects the effectiveness of the ATSPPF module and the network structure we have designed from another perspective.

In order to validate the generalization performance of our method, we performed training on the publicly available dataset NEU-DET [41] and compared the experimental results. The results are shown in Table 7. The NEU-DET dataset has more complex defect types and is more difficult to detect. Although Yolov3-tiny has a similar structure to our method, Table 6 shows that our method has both 19.4% and 15% higher indicators in ${mAP}_{0.5}$ and ${mAP}_{0.5–0.95}$ than Yolov3-tiny. In addition, it performs better on this dataset than other networks. This indicates that our approach demonstrates superior performance.

## 5. Conclusions

Wire bonding defect detection after chip packaging is essential to reduce production costs, improve chip life, and ensure the normal operation of equipment. Therefore, this paper proposes an automatic real-time chip detection method based on a convolutional neural network. We designed two modules: the CGC and ATSPPF modules, and based on the highly efficient network structure of yolov3-tiny, we have proposed an ATNet defect chip detection network using these two modules. The experimental results indicate that the mAP_0.5_ and detection speed of the ATNet method are 99.4% and 146 fps, respectively, which are much higher than our baseline yolov5. The GFLOPs and model size were also reduced by 48.75% and 35.82%, respectively, and the detection speed has seen a near 50% improvement. At the same time, for the mAP_0.5_, recall rate, and recognition accuracy, ATNet converges faster than yolov5s. In the future, we will continue to optimize the algorithm to achieve higher accuracy, faster detection speeds, and lower model complexity.

## Figures and Tables

**Table 1:** Five chip defect types and the number of each type of defect collected.

Sample	** 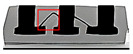 **	** 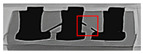 **	** 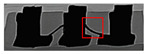 **	** 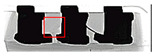 **	** 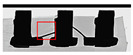 **
Number	712	724	642	664	656
Type	high loop	broken wire	low loop	wire missing	sagged wire

**Table 2:** Detailed architecture of ATSPPF.

Layer Name	Filters	Output Shape
CBL	128	20 × 20 × 128
MaxPool (k = 5)	/	20 × 20 × 128
MaxPool (k = 9)	/	20 × 20 × 128
MaxPool (k = 13)	/	20 × 20 × 128
Concat	/	20 × 20 × 512
CBL	256	20 × 20 × 256
CBAM	256	20 × 20 × 256
Concat	/	20 × 20 × 512
CBL	256	20 × 20 × 256
Add	/	20 × 20 × 256

**Table 3:** Ablation study of ATNet.

Number	C3	CGC	ATSPPF	${mAP}_{0.5}$	mAP_0.5–0.95_	FPS
1	✓	—	—	0.992	0.677	142
2	—	✓	—	0.993	0.687	151
3	✓	—	✓	0.989	0.695	97
4	—	✓	✓	0.994	0.693	146

**Table 4:** Comparison of the effects of different attention mechanisms in the ATSPPF module.

SE	CA	CBAM	${mAP}_{0.5}$	mAP_0.5–0.95_
✓	—	—	0.992	0.692
—	✓	—	0.992	0.687
—	—	✓	0.994	0.693

**Table 5:** The recognition results in comparison to the state-of-the-art methods on the WBD dataset.

Method	${mAP}_{0.5}$ (%)	FPS	GFLOPs (G)	Params (M)
Faster R-CNN-ResNet50	95.8	23	250.0	108.0
Dynamic R-CNN-ResNet50	95.9	21	248.5	107.0
RetinaNet-ResNet50	95.9	28	227.9	93.4
SSD300-VGG16	94.4	56	30.8	92.5
VFNet-ResNet50	95.1	21	224.5	98.3
Yolov5n	98.8	105	4.5	1.9
YOLOv3	98.8	64	155.0	117.0
YOLOv3-tiny	99.2	143	13.0	8.28
YOLOv5s	99.1	96	16.0	7.2
YOLOXs	97.1	56	13.2	8.5
GhostNet-YOLOv5s	99.0	53	8.3	5.4
ATNet(ours)	99.4	146	7.2	3.97

**Table 6:** The recognition results in comparison to the state-of-the-art methods.

	Method	Backbone	Recall	Precision	${mAP}_{0.5}$	${mAP}_{0.5–0.95}$
Original	Yolov3	\	0.988	0.993	0.988	0.703
	Faster-rcnn	resnet50	0.990	0.984	0.958	0.692
	SSD	vgg16	0.938	0.952	0.944	0.681
	SSD	mobilenetv2	0.942	0.957	0.935	0.686
	YoloR	\	0.965	0.956	0.961	0.689
	LFyolo	\	0.981	0.978	0.971	0.699
	Sclaed_yolov4	\	0.931	0.956	0.959	0.708
With ATSPPF	Yolov3	\	0.996	0.997	0.992	0.719
	Faster-rcnn	resnet50	0.987	0.987	0.965	0.698
	SSD	vgg16	0.945	0.966	0.957	0.690
	SSD	mobilenetv2	0.954	0.953	0.942	0.692
	LFyolo	\	0.959	0.966	0.984	0.696
	YoloR	\	0.987	0.982	0.981	0.709
	Sclaed_yolov4	\	0.961	0.966	0.976	0.711

**Table 7:** Comparison of recognition results for the NEU-DET dataset with state-of-the-Art methods.

Method	${mAP}_{0.5}$ (%)	${mAP}_{0.5–0.95}$ (%)	GFLOPs (G)	Size (M)
YOLOv5-mobilenetv3	67.6	32.6	11.3	13.8
YOLOxs	63.3	31.2	13.2	68.5
SSD300-VGG16	67.6	29.6	30.8	186.0
YOLOv7-tiny	65.5	29.1	13.2	12.3
YoloR	61.8	26.2	80.7	141.0
LF-YOLO	59.5	23.4	16.3	14.9
PicoDst-s [42]	59.1	24.3	13.8	3.1
Yolov3-tiny	52.1	20.2	13.0	17.5
YOLOv5s	69.8	32.5	16.0	6.7
ATNet(ours)	71.5	35.2	7.2	8.5

## Data Availability

The raw/processed data and modeling codes required to reproduce these findings cannot be shared at this time as the data also forms part of an ongoing study.
